# Mapping intended spinal site of care from the upright to prone position: an interexaminer reliability study

**DOI:** 10.1186/2045-709X-22-20

**Published:** 2014-05-16

**Authors:** Robert Cooperstein, Morgan Young

**Affiliations:** 1 Palmer West College of Chiropractic, 90 East Tasman Drive, San Jose, CA 95134, USA

## Abstract

**Background:**

Upright examination procedures like radiology, thermography, manual muscle testing, and spinal motion palpation may lead to spinal interventions with the patient prone. The reliability and accuracy of mapping upright examination findings to the prone position is unknown. This study had 2 primary goals: (1) investigate how erroneous spine-scapular landmark associations may lead to errors in treating and charting spine levels; and (2) study the interexaminer reliability of a novel method for mapping upright spinal sites to the prone position.

**Methods:**

Experiment 1 was a thought experiment exploring the consequences of depending on the erroneous landmark association of the inferior scapular tip with the T7 spinous process upright and T6 spinous process prone (relatively recent studies suggest these levels are T8 and T9, respectively). This allowed deduction of targeting and charting errors. In experiment 2, 10 examiners (2 experienced, 8 novice) used an index finger to maintain contact with a mid-thoracic spinous process as each of 2 participants slowly moved from the upright to the prone position. Interexaminer reliability was assessed by computing Intraclass Correlation Coefficient, standard error of the mean, root mean squared error, and the absolute value of the mean difference for each examiner from the 10 examiner mean for each of the 2 participants.

**Results:**

The thought experiment suggesting that using the (inaccurate) scapular tip landmark rule would result in a 3 level targeting and charting error when radiological findings are mapped to the prone position. Physical upright exam procedures like motion palpation would result in a 2 level targeting error for intervention, and a 3 level error for charting. The reliability experiment showed examiners accurately maintained contact with the same thoracic spinous process as the participant went from upright to prone, ICC (2,1) = 0.83.

**Conclusions:**

As manual therapists, the authors have emphasized how targeting errors may impact upon manual care of the spine. Practitioners in other fields that need to accurately locate spinal levels, such as acupuncture and anesthesiology, would also be expected to draw important conclusions from these findings.

## Introduction

Manual therapists use a great variety of physical examination methods to identify the optimal site of care for spinal complaints. Examiners have generally focused on joint malposition and joint hypomobility, or static and dynamic findings of abnormality, respectively. In addition, some contemporary practitioners have emphasized so-called provocation listings, which entail determining the impact of provocative interventions. These include changes in patient body position, orthopedic testing, joint challenges, and contacts with putative reflex points of various kinds [1]. Mnemonics such as P.A.R.T.S. [2,3] in chiropractic and TART [4,5] in osteopathy conveniently categorize the examination methods used by manual therapists. As convenient as such categorizations may be to inventory and assay manual therapy examination methods, they do not capture how the procedures are performed nor what differences may exist among practitioners methods of deployment. (Table 1 lists the components of P.A.R.T.S. and TART). As a case in point, neither P.A.R.T.S. nor TART specifies the *position* in which the patient is assessed. Variations in the patient positioning procedure may lead to very different conclusions about the optimal site of care, the vector, and the other force characteristics deployed in spinal manipulation and other interventions. This may impact upon the reliability, validity, and ultimately clinical utility of the examination procedure.

**Table 1:** Acronyms used in manual therapy to summarize examination procedures

**PARTS acronym (chiropractic) **[2]**,**[3]	**TART acronym (osteopathy) **[4]**,**[5]
***P*** ain: location, quality, intensity of pain or tenderness	***T*** issue texture changes: temperature, tense, flabby, edematous, fibrotic, indurated, hypertonic, hypertrophied
***A*** symmetry: of sectional or regional spinal components identified by static palpation of structures	***A*** symmetry in joint motion; e.g., compared with contralateral tissues
***R*** ange of motion: Decrease or loss of specific active or passive movements identified by motion palpation	***R*** estriction of joint motion: degree and quality of pliability, mobility, stability; quality of end-feel
***T*** one, ***T*** exture, ***T*** emperature: changes in specific soft tissues identified through palpation	***T*** enderness (or sensitivity) of soft tissues: unnaturally sensitive, tender, painful, numb with pressure or on active/passive movement
***S*** pecial tests: linked to proprietary technique systems, not in general use	

As an example, there is some evidence that supine measurements of cervical rotational range of motion are not correlated with seated measurements [6-9]. Notwithstanding the gross similarity of supine and seated measuring methods, seated weight-bearing and supine non-weight bearing measures of range of motion apparently assess different clinical phenomena. This could lead to different clinical interventions. As another example of patient position-dependent examination findings, the relative position of the posterior superior iliac spines depends on whether the patient is standing or upright. Asymmetry seen in the standing position might be different and even reversed in the seated position [10]. As a final example of how patient position impacts physical examination findings, the position of the inferior angle of the scapula (IAS) in relation to the spine varies between the upright and prone positions; and also depends on the patient’s arm position [11-13]. Furthermore, the scapula tends to be relatively inferior on the side of the dominant arm [14]. These scapular anatomic findings and their clinical implications became the impetus for this study.

Although it is widely believed that on average the IAS is at the T7 spinous process (SP) when upright, and the T6 SP when prone, there are data in the manual therapy literature suggesting otherwise [11-13]. Table 2 compares the conventional wisdom (“7 up, 6 down”), with evidence-based findings (“8 up, 9 down”) on the location of the IAS in relation to the SPs. The position of the IAS also varies according to the patient’s arm positions on the table [12]. The data show a range of T6-T10 for the standing scapular tip, and T7-T11 for the prone scapular tip [11-13].

**Table 2:** Inferior scapular angle and spinal landmark association

	**Spinal level, conventional wisdom**	**Spinal level, evidence-based**
Upright IAS	T7	T8
Prone IAS	T6	T9

Three studies by anesthesiologists came to similar conclusions. Using a radiological reference standard, Teoh [15] found the IAS in the standing position to be on average nearest to the T8 SP, as did Arzola [16] using a portable ultrasound system reference standard. Kim [17], using a radiological reference standard, found the scapular tip usually lined up with the T7 rather than T8 SP as in the other 2 anesthesiology studies. The discrepancy is only in appearance, since Kim’s data were gathered in the “epidural position” (seated, back arched, neck flexed, arms across the chest), which is known to raise the scapular tip by one level compared to where it lies in the “anatomical position” (thorax fully upright, palms anterior). Thus, Kim’s data for the upright position can be easily transformed to upright findings by simply subtracting one vertebral level from each reported data point, rendering this researcher’s data consistent the other anesthesiology studies. Ernst [18], using cross-comparison with other manually palpated anatomical landmarks, also reported the IAS to correspond to the T8 SP.

The purpose of the present study was two-fold, resulting in two experiments. In the first, the authors derive conclusions based on scapular landmarks for the practice of manual therapy and other professions (including nursing, surgery, anesthesiology, and acupuncture). In the second experiment, the authors propose and determine the reliability of a novel procedure for mapping spinal sites initially identified upright to the prone position. The overarching goal was to improve upon scapula-based landmark protocols for localizing spinal landmarks that are position-dependent, thus reducing the likelihood of targeting errors.

There has been some assessment of the reliability of static palpation, but these did not explore any issues pertaining to patient-position variations. A systematic review of static palpation of the spine and sacroiliac joints [19] identified 14 studies focused on locating painful or tender points, 10 on the location of landmarks, and 5 on position or alignment of bone structures. Palpation for pain was more reliable than palpation for position, for which examiner concordance was low. A systematic review of the methods used by manual therapists to identify the optimal site for spine care came to the following conclusion for static palpation: “Based on high quality evidence, the validity of palpation for localizing the site of care is unclear” [20]. Neither of these 2 static palpation reviews addressed the complications of using landmarks in the upright position to determine care to be rendered prone.

### Experiment 1: Estimating the magnitude of targeting errors

To determine the magnitude of targeting errors attributable to incorrect scapular landmark rules, the authors conducted a *Gedankenexperiment*, or “thought experiment”. Thought experiments explore the potential consequences of a principle or hypothesis, sometimes where the outcome is so intuitively obvious that a physical demonstration of this same principle could or would be deemed superfluous.

The magnitude of spinal targeting errors due to using an inaccurate scapular landmark rule may be heuristically calculated by working out two examples, one for radiographic and the other for physical examination procedures. There would be errors in both the site of care and numerated level of care that is charted. In these examples, to simplify the discussion, it is assumed that the IAS is even with a specific vertebral level; even though the IAS lies within a range, as discussed above. Equally discounted is the fact that that the IAS may not lie at the same level on the left and right sides of the body, and would be expected to be relatively inferior on the side of the dominant arm [14].

### Example 1: Upright radiographic examination

Suppose a radiograph is being scrutinized to determine a spinal level of interest warranting clinical intervention. (It is acknowledged that the use of radiographic examination for biomechanical analysis is controversial, but that is beyond the scope of this project). Having directly and hypothetically correctly identified T7 on a radiograph, the clinician now attempts to identify the prone T7 SP for the purpose of a manipulative or other manual procedure. If the clinician uses the (inaccurate) landmark rule that the IAS is at the T6 SP while prone, with hands placed on the arm rests, an adjustive contact is made one level lower than the IAS. Since in reality the IAS is at the T9 SP prone, [12] counting down one spinal level results in the clinician contacting T10, not the intended T7 level. Thus, there is a 3-level targeting error. There would also be a 3 level charting error in numerating the site of care, since T7 was the intended but T10 the actual site of care.

### Example 2: Upright physical examination

The targeting error for other forms of upright patient assessment is less severe, at least for treatment if not charting. Let us assume that upright (sitting or standing) thermography, manual muscle testing, or motion palpation identifies a vertebral site of care whose SP is at the ISA. Using the traditional benchmark rule, the clinician believes this to be T7, whereas in fact it is more likely closer to T8. If the clinician uses the (inaccurate) landmark rule that the IAS is at the T6 SP while prone, with hands placed on the arm rests, an adjustive contact is made one level lower than the prone IAS. Since the it is actually at T9, counting down one level identify T10. Thus there is a 2-level targeting error: the clinician treats T10 rather than T8, the actual intended site of care. Since the clinician believes this to the T7 level, there would be a 3 level charting error in numerating the site of care, the same degree of charting error made in the case of radiographic examination.

Although these examples assumed findings at either T7 or T8, if the clinician applies the “7 up, 6 down” rule, findings in other thoracic locations would be associated with the same 3 or 2 level targeting error (for radiographic and other physical examination procedures, respectively). In this highly simplified experiment, the authors did not consider the possibility of examiner errors other than those accruing to the use of an erroneous landmark rule; nor did they explore the consequences of variation in scapular positions among between patients, nor within patients between the left and right sides of the body.

### Experiment 2: The reliability of an alternative targeting “SP-to-prone” targeting procedure

Since the application of inaccurate scapular benchmark rules likely results in targeting errors for the site and numeration of spine care, it would be useful to devise a more accurate way to identify a prone site of care based on upright examination findings. Experiment 2 was based on the hypothesis that palpators could identify a spinal site of care landmark in the upright patient and then reliably track that site to the prone position. The described procedure requires maintaining contact with a SP as a patient moves from the upright to the prone position; for this reason the authors created the descriptive term “SP-to-prone” to name the procedure.

## Methods

**Figure 1:**
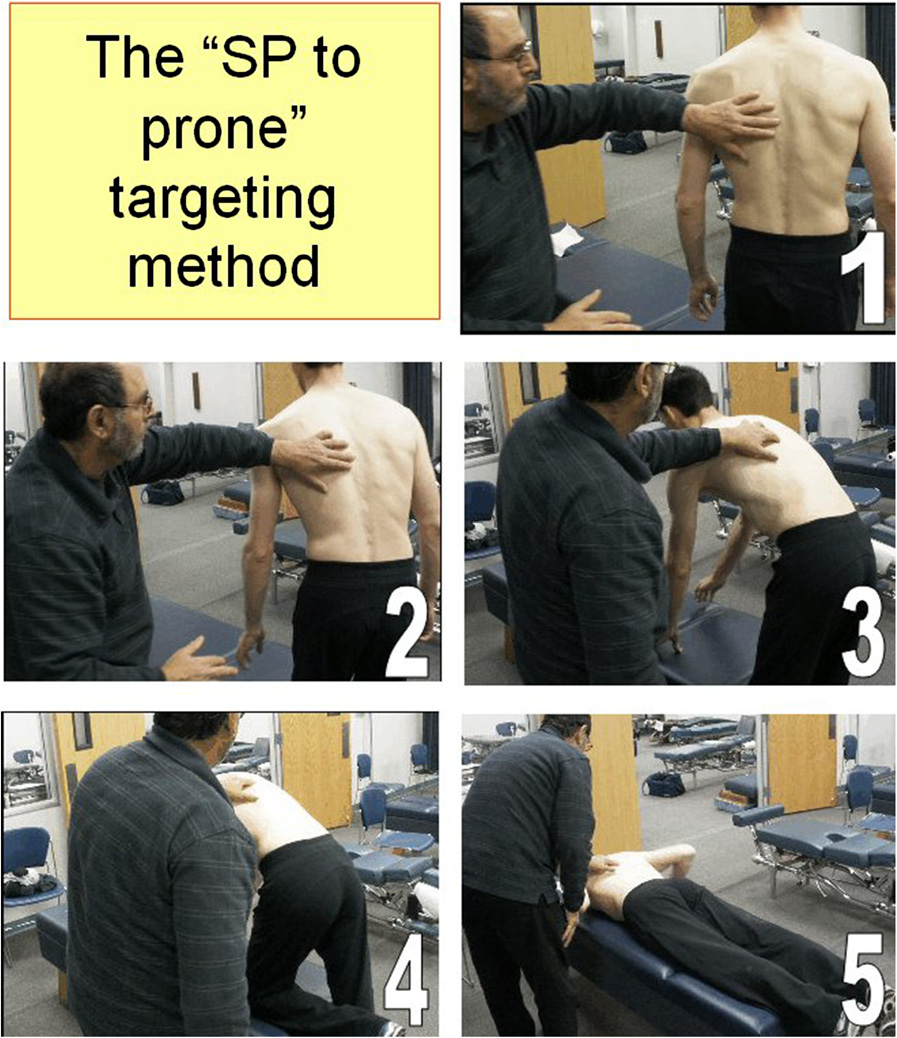
**The SP-to-prone targeting procedure.**

**Figure 2:**
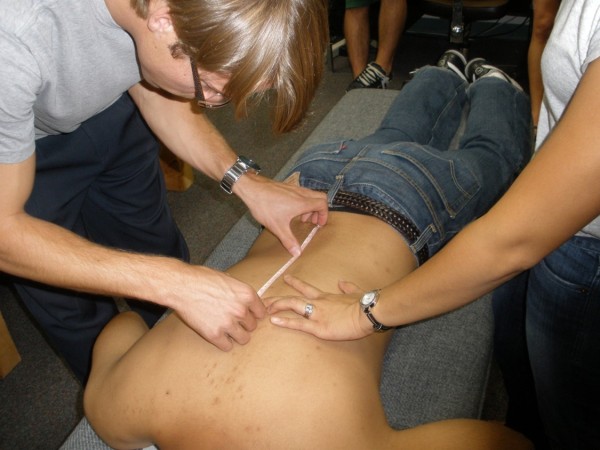
**Determining distance of palpatory end-point to lumbar skin mark.**

There were 2 experienced examiners in this study, with many years of teaching experience and clinical practice and 8 novice student examiners. The first author was experienced in the SP-to-prone method in a private practice setting for several years. The second author has taught palpation at a chiropractic college and practiced for several years. There were no practice or rehearsal sessions for this study. It was approved by the Palmer Chiropractic College Institutional Review Board and the 2 participants provided written consent. They were a convenience sample of asymptomatic young men. A very small mark using an erasable marker was placed on the skin at the SP most closely even with the inferior tip of the right scapula in the standing position. Another mark was placed on the SP that was most closely even with the iliac crest, at either L4 or L5 [21]. Then, standing somewhat behind the participant and to his left, each palpator positioned the distal tip of the left middle digit parallel to the skin mark, trying as closely as possible to align the skin mark with the middle of the nail, as indicated with a penciled line parallel to the length of the line. With the fingernail in position the skin mark was not visible. The palpator kept the palpating finger firmly on the SP underlying the skin mark while the participant carefully and deliberately repositioned himself on the table in the prone position (Figure 1). A study assistant then measured the distance in mm from the lumbar marker to the middle of the nail, which was kept parallel to the participant’s mid-thoracic spine (Figure 2). Each of 10 palpators performed this protocol on each of the two participants, in the same order for each participant. Approximately 2 minutes elapsed between observations. The examiners waiting to be tested were at least 5 meters away from the location where the measurements were being recorded and could not have visually ascertained the site localized in the prone position.

Statistical analysis included calculating the absolute value of the mean difference for each examiner from the 10 examiner mean for each of the 2 participants (MAD), the standard error of the mean (SEM), the root mean square error (RMSE, or σ_e_), and the intraclass correlation coefficient (ICC). Some of the analyses were performed using Excel® 2013(Microsoft Corporation) and some with SPSS® v.19 (IBM Corporation).

## Results

The data are summarized in Table 3, all distances in millimeters. For participant 1, the average distance from lumbar to thoracic skin marker was 243.4 mm; and for participant 2, was 221.4 mm. The ranges for examiner errors (differences in mm for each measurement from the 10 measure mean) were -8.4 to 11.6, and -6.4 to 10.6, for participants 1 and 2 respectively. The mean examiner error for 20 observations ≈ 0. Stratified by experience and averaged for the two participants, MAD values for the 8 novice examiners and 2 experienced examiners were 4.4 and 6.7 mm, respectively. The 10 examiner MAD for each of the 2 participants = 5.2 mm for participant 1, and 4.5 mm for participant 2; an average of 4.7 mm. Since the computed t value was less than the t-critical value, the null hypothesis that MAD was not different from zero at a 95% confidence level was not rejected. Standard error of the mean (SEM) for all examiners and participants = 0.8 mm, and root mean square error (RMSE, or σ_e_) = 5.9 mm. For all 10 examiners and participants, ICC (2,1) = 0.83, which is judged “excellent” according to the Landis scale [22].

**Table 3:** Results

**(mm)**	**Participant 1**	**Participant 2**	
Distance IAS-SP to L4 SP	243.4	221.4	
Range, examiner errors	-8.4, 11.6	-6.4, 10.6	Mean
MAD exp (n = 2)	7.4	6.0	6.7
MAD non-exp. (N = 8)	4.7	4.1	4.4
			Participants combined
MAD all, n = 10 95% CI: (lower, upper)	5.2 (2.7, 7.7)	4.5 (2.7, 6.3)	4.8 (3.3, 6.4)
SEM	1.3	0.9	0.8
RMSE (σ_e_)	6.5	5.3	5.9
ICC (2,1)	0.83		

*MAD* = mean absolute difference; *SEM* = standard error mean; *CI* = confidence interval; *ICC* = intraclass correlation coefficient; σ_e_ = root mean square error (*RMSE*); *SEM* = (MAD SD / √N; 95% *CI* = SD ± SE*1.96; *RMSE* (σ_e_) = √MSE.

## Discussion

Manual therapists often examine the patient in a position different from the one in which the patient will be treated. Thermographic assessment [23], manual muscle testing for spine dysfunction [24], and spinal motion palpation [25] are usually performed with the patient upright, but often lead to spinal interventions, including but not limited to manipulation, that are performed on a prone patient. Since chiropractors usually take or order upright x-rays, radiographic examination is also upright yet may lead to treatment in a prone position. Where is the guarantee that vertebral sites identified as areas of concern in the upright position can be reliably palpated and treated in the prone position? It seems that the ability of manual therapists to accurately proceed from upright examination findings to prone interventions has been simply assumed. Targeting errors could in turn lead to sub-optimal clinical outcomes, depending on the degree to which specificity in identifying sites of spine and sacroiliac care is clinically important. These targeting errors could also lead to charting errors, in that the *intended* sites of care and/or *actual* sites of care could be incorrectly numerated. Byfield and Kissinger, addressing the issue of errors in identifying spinal segmental levels, consider the impact of problems in locating anatomical landmarks due to tissue movement, examiner bias, and recording mistakes. This, they write, could “pose potential problems when palpatory findings are compared from one palpating posture to another” ([26], p.19). These authors also draw a distinction between end play or end feel palpated in the seated position, and joint play or challenge that is palpated prone both assessed at the endpoint of passive spinal motion, at the elastic barrier of the joint ([26], p.10).

The thought experiments suggested that targeting errors using the IAS to locate spinal levels identified in the upright position on a prone patient would be substantial, ranging from 2 to 3 spinal levels. To address that problem, the first author devised a novel method which attained substantially perfect reliability and probably accuracy, among both experienced and novice palpators, in mapping upright levels to the prone position. The authors proposed that the described SP-to-prone targeting method could be utilized in any patient population in which potential spinal sites of care need to be mapped from upright to prone position. Since the reliability experiment was construed to be an IRB-approved full-dress rehearsal for a future larger study, it was not considered important to perform a power analysis to decide upon the numbers of participants and examiners. When it became clear during data analysis that the reliability was extremely high, with very modest variability in examiner errors, it was decided that a larger study was not required. In small studies with relatively few participants, the number of examiners is also commensurately few [27].

Although the length of the thoracic spine varies between individuals, on average it is about 28 cm in length [28], which corresponds to about 23.3 mm per level. Therefore, with a range of -8.4 to 11.6 mm for examiner differences from the 10 measurement mean for each of 2 participants, none of the 20 measurements from the L4 SP to the targeted thoracic skin marker were discrepant by more than half a vertebral level (approximately 11.7 mm). In fact, the 20-measurement MAD = 4.8 mm was less than 1/4 the length of a typical thoracic spinal level. Therefore, the examiners in this study should be considered to have been 100% successful in their effort to maintain contact on the same spinal level as the participants moved from the standing to the prone position. The square root of mean squared error (MSE) yields root-mean-square error (RMSE), yet another measure of examiner accuracy. In this study, combined RMSE = 5.9 mm, while combined (10 observers, 2 participants) MAD = 4.8 mm. MAD = RMSE when all the errors are of the same magnitude. With increasing variance in the errors, RMSE increases in relation to MAD, and thus becomes a more useful (certainly a more conservative) estimate of accuracy. These computations confirm the examiners most likely identified the same spinal level in the prone position in all cases. With ICC (2,1) =0.83, using the Landis scale [22] for classifying ICC values (below 0.40 = poor, 0.40-0.59 = fair, 0.60-0.74 = good, above 0.75 = excellent) interexaminer agreement on the prone spinal level corresponding to the upright thoracic skin marker was judged to be “excellent”.

By design, experiment 2 only addressed interexaminer reliability, not the validity (i.e., accuracy) of the attempt to identify the same segment in the upright and prone positions. Since there was no reference standard in this study, the authors thought it reasonable to use the 10 examiner mean for the prone targeted level as a surrogate for such a reference standard. Examiners may highly agree with each other, and yet be inaccurate – that is, they may both be wrong. However, the authors think it highly unlikely that the palpators in this study were subject to systematic measurement error, such as may have resulted in all 10 palpators identifying the same wrong level in the prone position. This is due to the fact that the mean examiner error ≈ 0, ruling out systematic bias. The results using mostly novice examiners most likely represents a worst case scenario, since one would expect more experienced examiners who might use the SP-to-prone procedure in actual clinical situations to achieve even higher accuracy. Therefore, the excellent interexaminer reliability achieved with an examiner pool in which 8/10 (80%) were novice palpators bodes well for clinical relevance.

To the extent clinicians in the manual therapy professions feel that spinal level specificity improves the outcome of interventions, they may find it worthwhile to use the described SP-to-prone method as an alternative to the more usual but suspect scapula landmark when they need to locate spinal levels identified in the upright position on a prone patient. As an alternative, they could use some other landmark to locate a prone spinal level, such as the iliac crest (thought to be in line with the SP of L4 or L5 [21] or the vertebra prominens (VP), thought usually – but not always – to line up with C7 [29]. Due to the variability of the L4 = iliac crest and VP = T1 landmark rules, the authors think the upright-to-prone targeting method herein described would be more reliable. Although it enables reliably mapping upright physical examination finding to the prone position, it does not facilitate accuracy in charting. Indeed, that would have to be accomplished using some other counting method, one that best not involve any scapular landmark rules. Although the SP-to-prone targeting method most directly supplants using landmarks to target a prone spinal level base on upright *physical* examination, it is unlikely to reduce targeting errors accruing to *radiographic* examination.

Beyond the manual therapy professions, these findings may be relevant to other professions that commonly use the IAS as a landmark to target spinal levels. Practitioners of orthopedic medicine, neurology, nursing, and acupuncture should also be interested in this updated information on the anatomical relation of the scapula and spine. For example, anesthesiologists require precise placement of thoracic epidural catheters to optimize postoperative analgesia and minimize adverse effects [15,17,30]. Anatomic landmarks are also used to locate acupuncture points, and specifically the IAS has been cited as a reference point for the T7 spinous process ([31], p.11). Surgeons may decide upon a location to begin their incision based at least in part on the location of the IAS ([32], p18, 26).

In manual therapy, the preponderance of research justifies considerable confidence in treatment success, but not nearly as much in diagnostic acumen. This creates a challenging paradox. It is widely believed that successful treatment requires an accurate diagnosis. If so, it is difficult to explain the apparent success of a great variety of manual treatment procedures, when demonstrated low levels of interexaminer reliability in widely used examination procedures suggests the notion of a specific chiropractic diagnosis (i.e., “listing”) remains somewhat hypothetical. As an example, in the derivation of a clinical prediction rule for spinal manipulation, not one of 18 sacroiliac orthopedic tests were retained in the final model that predicted clinical success [33], even though the manipulative procedure used in the study was intended for the sacroiliac joint.

### Limitations of experiments 1 and 2

Experiment 1 is a *Gedankenexperiment*. A thought experiment is only capable of attaining face validity, deriving conclusions that seem to make sense. Not all such experiments have good outcomes, such as the one prior to the time of the ancient Greeks (and still widely embraced up to the time of Columbus) by which visual cues led to the conclusion that the earth was flat.

In experiment 2, the investigators did not attempt to numerate the spinal level of the skin marker that was parallel to the IAS, nor how much it changed between the upright and prone levels. Since that had been done in previous studies [11-13], this study was confined to targeting reliability. Since the authors were not aware of what proportion of clinicians actually uses the scapula to localize spinal sites as compared with other landmarks, they did not have an estimate of the magnitude of the problem being addressed. That stated, the authors do believe this proportion is high, certainly for localizing thoracic spine sites.

In principle an examiner could have deceitfully moved the index finger he or she was attempting to keep on a skin mark (concealed under the fingernail) had he or she seen the index finger slip to such a degree that the skin mark could be seen as the participant assumed the prone position. The examiners were asked to look away from their index fingers to preclude that opportunity, and in no cases did the research assistant who recorded the distance of the endpoint from the lumbar skin mark report that the thoracic skin mark was no longer occluded by the distal index finger.

This study did not address the *clinical importance* of targeting specificity for manual therapists. A comprehensive systematic review [20], while it describes the methods that are typically used by chiropractors to target sites of spinal care and rates their validity and reliability, does not address the clinical utility of this information. Although studies in recent years have questioned the ability to deliver forces to intended targets [34-42], it is not known to what extent diagnostic and/or adjustive imprecision in the manual therapy professions leads to suboptimal clinical consequences. The authors would be outside their scope of practice to comment on to what extent targeting errors impact clinical outcomes in the other health care professions that use spinal landmarks.

Experiment 2, the reliability study, was intended to be a pilot study to test the methodology. When the data were analyzed and showed very high levels of examiner concordance, it became clear that a larger study was not necessary. This explains why there were only 10 examiners and 2 participants in this study, resulting in only 20 observations. Caution should be exercised in extrapolating the results to diverse patient populations who might differ significantly from the 2 healthy young male participants in this study. Symptomatic patients, were they to exhibit awkward movements as they change from the upright to the prone position, could make it more difficult to maintain contact on the targeted spinous process, threatening accuracy.

## Conclusions

This is a deceptively simple study that addresses a matter that in the authors’ opinion is very important, and yet is rarely discussed let alone studied: how does a manual therapist know that a spinal site identified as worthy of treatment with the patient in the upright position has been accurately located when the patient has moved to the prone position? The ability to be accurate in this endeavor using spinal landmarks such as the IAS, crest of ilium, and vertebral prominens has been more or less assumed. It would be presumptuous to assume this in turn has rendered clinical outcomes suboptimal and yet that is clearly possible. Even in the case there had been no deleterious impact on the quality of care, it would then seem that the information supplied by typical spinal examination procedures would have been of little import. That would certainly be worth knowing. As manual therapists, the authors have emphasized how targeting errors might impact upon manual care of the spine. Practitioners in other fields that need to accurately locate spinal levels, such as acupuncture and anesthesiology, would also be expected to draw important conclusions from these findings.

The clinical rationale for insisting on a high degree of diagnostic specificity, the ability of examiners to agree on an optimal site and vector for care, and their ability to direct an intervention accurately at an intended site of care, are far from established. Despite the ample evidence that manipulative and other manual spinal interventions are both safe and effective, the degree to which the results of clinical studies would change were targeting practices more accurate remains speculative. The authors suggest it would be easier research the possibility of optimizing the site of spinal care were there reliable methods to track spinal sites of interest identified in the upright position to the prone and other positions; that is the spirit in which the SP-to-prone targeting procedure has been proposed.

## Competing interests

The authors declare they have no competing interests.

## Authors’ contribution

RC developed the targeting procedure that is the focus of this study, participated in data collection, and drafted the first draft of the manuscript. MY participated in data collection and the production of the final manuscript. Both authors read and approved the final manuscript.
